# The role of sodium pyruvate in mitigating the cytotoxic effects of vanadium on CHO-K1 cells

**DOI:** 10.1038/s41598-025-09606-7

**Published:** 2025-07-05

**Authors:** Iwona Zwolak, Ewa Wnuk, Elżbieta Kochanowicz

**Affiliations:** 1 Department of Biomedicine and Environmental Research, Institute of Biological Sciences, Faculty of Medicine, The John Paul II Catholic University of Lublin, Konstantynów Ave. 1J, 20-708 Lublin, Poland; 2 Department of Molecular Biology, Institute of Biological Sciences, Faculty of Medicine, The John Paul II Catholic University of Lublin, Konstantynów Ave. 1I, 20-708 Lublin, Poland

**Keywords:** Vanadium, Pyruvate, Antioxidant, Metal, Oxidative stress, Apoptosis, Toxicology, Metals

## Abstract

Vanadium is a hazardous, pro-oxidant element that contributes to environmental pollution and has been reported as a risk factor for human health through occupational or environmental exposure. Pyruvate, on the other hand, is a natural alpha-keto acid with exceptional antioxidant and cytoprotective properties. Therefore, the aim of this study was to evaluate the mitigating effect of exogenous pyruvate against vanadium-induced toxicity in cultured Chinese hamster ovary (CHO)-K1 cells. To this end, CHO-K1 cells were exposed to 100 μM vanadyl sulfate (VOSO_4_) for 24 h in the presence of 4.5 and 8 mM sodium pyruvate. Cell proliferation and morphological changes, cellular ATP levels, antioxidant stress (GSH) levels and apoptosis markers (caspase 3, 9, annexin V binding) were assessed to investigate the effect of sodium pyruvate on VOSO_4_-induced damage in CHO-K1 cells. The results showed that VOSO_4_ induced morphological changes, inhibited cell proliferation, decreased cellular ATP and reduced glutathione levels. Co-treatment of VOSO_4_-intoxicated CHO-K1 cells with sodium pyruvate significantly reduced these cytotoxic effects. Analysis of apoptosis and necrosis showed that VOSO_4_ slightly induced apoptosis and necrosis, and exogenous pyruvate inhibited the cytotoxicity of the tested vanadium dose in CHO-K1 cells, mainly by reducing the necrosis effect. The cytoprotective effect of exogenous pyruvate was also confirmed in normal mouse fibroblast (NIH/3T3) cells demonstrating that the protective properties of pyruvate are not cell specific.

## Introduction

Vanadium is a heavy metal that occurs naturally in minerals, coal and oil. The metal is widely used in industry, particularly in metallurgy for the production of steel. However, vanadium is also classified as a hazardous pollutant and its accumulation in the environment including air and soil has increased in recent years^1–3^. The main anthropogenic sources contributing to environmental pollution by this metal are the extraction and combustion of fossil fuels (coal and oil), steel production, the chemical industry and the production of redox batteries^4^. Vanadium enters the body through the lungs or the digestive system. It is transported with the blood in the form of vanadyl or vanadate in combination with transferrin, among others, to other tissues, including bones, which are the main reservoir of vanadium in vertebrates^5,6^. Harmful effects of vanadium reported from occupational exposure to dusts containing the metal include respiratory symptoms in boiler workers repairing oil-fired boilers^7^ and neurobehavioural changes in metal workers^8^. In addition, epidemiological studies have also suggested links between environmental vanadium and respiratory disease in children in New York, USA^1^ and Vilnius, Lithuania^9^, as well as adverse effects of vanadium on pregnancy development^10^ and cardiovascular disease^11^ in Chinese studies. In addition, vanadium in the form of V_2_O_5_ is classified by the International Agency for Research on Cancer (IARC) as a Group 2B carcinogen^12^.

Vanadium exposure and toxicity have been associated with the induction of pro-oxidant, pro-inflammatory and pro-apoptotic effects in both in vivo studies^13–15^ and in vitro studies^14^. In fact, vanadium generates reactive oxygen species (ROS) by various mechanisms, mainly as a side-effect of the oxidation and reduction reactions that the metal undergoes in the presence of cellular oxidants and reductants, and vanadium-dependent mitochondrial dysfunction^16^. Furthermore, vanadium-dependent ROS generation may involve toxic changes and interactions between lysosomes and mitochondria, which further enhances vanadium-induced cell damage^17^. Excessive oxidative stress induced by vanadium leads to damage of proteins, lipids and DNA in the cell, resulting in cell death by apoptosis or necrosis. Due to the pro-oxidative mechanism of vanadium toxicity, natural antioxidants are considered effective vanadium detoxifying agents^5,18^.

Sodium pyruvate, the subject of this study, is known for its antioxidant, anti-inflammatory and cytoprotective properties in vitro^19,20^ and in vivo^21,22^ studies. The mechanism of the protective action of this simple α-keto acid is mainly due to its direct and very efficient decomposition of hydrogen peroxide (H_2_O_2_), which prevents oxidative stress^23–25^. An additional protective mechanism of pyruvate is its beneficial effect on mitochondrial function^26–28^. Given the strong antioxidant properties of pyruvate, the study was undertaken to assess the protective effects of this compound against vanadium cytotoxicity. Furthermore, the current study is a continuation of previous work by this research group^29,30^. The aim of this work is to verify whether the results obtained previously are constant and to obtain additional data regarding the protective properties of sodium pyruvate against vanadium cytotoxicity.

## Materials and methods

### Reagents

Dulbecco’s modified Eagle’s medium Hams F-12 (DMEM)/F12 1:1 (cat. No. D8437), fetal bovine serum (FBS, Cat No. F9665), bovine calf serum (CS, Cat No. 12138 C), L-glutamine solution (Cat No. G7513), antibiotic antimycotic solution (100 ×) (cat. No. A5955), DPBS (Dulbecco’s modified phosphate buffered saline), vanadyl sulphate hydrate (VOSO_4_·xH_2_O, cat. No. 204862), sodium pyruvate solution (100 mM, cat No. S8636), Trypsin solution (0.05%), In Vitro Toxicology Assay Kit, Resazurin based (cat. No. TOX8), Glutathione assay Kit, fluorimetric (cat No. CS1020), were purchased from Sigma-Aldrich (St. Louis, MI, USA).

CellTiter-Glo Luminescent Cell Viability Assay (G7570), Caspase-Glo® 3/7 Assay (cat No. G8091) and Caspase-Glo® 9 Assay (cat No. G8211) were from Promega (Promega Corporation, Madison, WI, USA).

Annexin V-FITC Apoptosis Assay was purchased from Immunochemistry Technologies, (Bloomington, MN, USA).

VOSO_4_·xH_2_O (assuming hydration of five molecules) was dissolved in deionised water to a final concentration of 10 mM stock solution (light blue in colour). The stock solution was prepared freshly every time just before experiments involving exposure of the cells to VOSO_4_.

### Cell culture

The CHO-K1 cell line (Chinese hamster ovary cell line K1, Sigma-Aldrich, ECACC 85,051,005) was cultured in DMEM/F12 1:1 containing 4 mM L-glutamine and 0.5 mM sodium pyruvate. The medium was supplemented with 5% (v/v) FBS and antibiotics (100 U/mL penicillin, 100 mg/mL streptomycin and 0.25 µg/mL amphotericin B).

The NIH 3T3 cell line (mouse Swiss NIH embryo cell line, Sigma-Aldrich, ECACC 93,061,524) was cultured in DMEM high glucose containing 4500 mg/L glucose and 1 mM sodium pyruvate. The medium was supplemented with 1% (v/v) L-glutamine and 5% (v/v) calf serum (CS). The CHO-K1 and NIH/3T3 cells were cultured in a humidified CO_2_ incubator at 37 °C. Cells were passaged three times per week with 0.05% trypsin solution.

### Resazurin-based cytotoxicity assay

In this assay, cytotoxicity is indicated by a decrease in the reduction of blue resazurin to its pink product resorufin. The amount of blue resazurin not converted to pink resorufin by the mitochondria of living cells is proportional to the number of damaged cells.

CHO-K1 cells were inoculated in 96-well plates and treated with 50 or 100 μM VOSO_4_ in the presence of 4.5 mM and 8 mM sodium pyruvate for 24 h. NIH 3T3 cells were seeded in 96-well plates and treated with 50 μM VOSO_4_ in the presence of 4.5 mM and 8 mM sodium pyruvate for 48 h.

After treatment of CHO-K1 and NIH 3T3 cells to the above concentrations of VOSO_4_ and sodium pyruvate, 10 μl of resazurin dye was added to each well and the cells were incubated at 37 °C for 3 h. The absorbance of unreduced resazurin was read at 600 nm in a microplate reader (Synergy 2, BioTek Instruments, Inc., Winooski, VT, USA); the reference wavelength is 690 nm. Resazurin assay data were expressed as percentage of control cells and calculated as follows: Atest/Acontrol × 100% (Atest: absorbance of cells treated with VOSO_4_ and/or pyruvate; Acontrol: absorbance of control cells). In this test, cytotoxicity (cell damage) is indicated by a percentage increase over control cells.

### ATP level measurement

ATP levels in CHO-K1 cells were measured using bioluminescence reactions based on the ATP-dependent oxygenation of luciferin to oxyluciferin by luciferase, resulting in light emission. The intensity of the luminescence signal is proportional to the amount of ATP present.

CHO-K1 cells were inoculated in a 96-well plate and treated with 100 μM VOSO_4_ in the presence of 4.5 mM sodium pyruvate. After 24 h, 100 μl CellTiter-Glo Reagent was added directly to each well, including the blank wells containing only culture medium (no cells). The plate was shaken on a microplate shaker for 2 min to induce cell lysis. After 10 min incubation at room temperature, the luminescence was read in a microplate reader against the blank (Synergy 2, BioTek Instruments, Inc., Winooski, VT, USA). Data were expressed as percentage of control cells and calculated as follows: LUtest-LUblank/LUcontrol-LUblank × 100% (LUtest: luminescence of cells treated with VOSO_4_ and/or pyruvate, LUcontrol: luminescence of control cells, LUblank: luminescence of blank wells).

### Mitochondrial ROS production measurement

Mitochondrial ROS production was detected using mitochondrial ROS detection reagent (cat No. 701600, Cayman Chemical, Ann Arbor, MI, USA) and MitoSOX Red (ThermoFisher, Eugene, OR, USA; M36008) which accumulates in the mitochondrial matrix and fluoresces in the presence of ROS.

CHO-K1 cells were inoculated into a 96-well plate and preloaded with Cayman’s mitochondrial ROS detection reagent for 20 min. Next, the cells were gently washed with DPBS (w/Ca/Mg) and treated with 100 μM VOSO_4_ in the presence of 4.5 mM sodium pyruvate. Fluorescence was read after 1 and 24 h incubation at 485/20 nm (excitation) and 590/35 nm (emission) in a microplate reader (Synergy 2, BioTek Instruments, Inc., Winooski, VT, USA). For MitoSOX Red staining, cells treated with 100 μM VOSO_4_ in the presence of 4.5 mM sodium pyruvate were stained for 15 min with 5 μM MitoSOX Red. Next cell media were removed and cells were gently washed with DPBS (w/Ca/Mg) and 200 μl DPBS was added to each well. Fluorescence was read at 360/20 nm (excitation) and 590/35 nm (emission) in a microplate reader. Data are expressed as a percentage of the control cells.

### GSH level determination

Reduced glutathione (GSH) levels were determined on the basis of the monochlorobimane probe (cat No. CS1020, Sigma-Aldrich), which binds to GSH in a reaction catalysed by glutathione S-transferase (GST) to form a fluorescent adduct. Briefly, CHO-K1 cells inoculated into a 96-well plate were treated with 100 μM VOSO_4_ in the presence of 4.5 mM sodium pyruvate. After 24 h, the cells were washed twice with DPBS and incubated with monochlorobimane for 20 min at room temperature. Fluorescence was then read at 360/40 nm (excitation) and 460/40 nm (emission) in a microplate reader (Synergy 2, BioTek Instruments, Inc., Winooski, VT, USA). Cellular GSH concentration was determined from the GSH standard curve and expressed as a percentage of control cells.

### Detection of caspase-3/7 and caspase-9 activity

Caspase-3/7 and caspase-9 activity in CHO-K1 cells was measured using bioluminescence reactions based on the caspase-dependent cleavage of the luminogenic substrate containing the tetrapeptide DEVD (caspase-3/7) or LEHD (caspase-9), resulting in the release of aminoluciferin, a luciferase substrate for light emitting catalysis. The intensity of the luminescence signal is proportional to the amount of caspase present.

CHO-K1 cells inoculated into a 96-well plate were treated with 100 μM VOSO_4_ in the presence of 4.5 mM sodium pyruvate. After 3 h of incubation, Caspase-Glo 3/7 reagent or Caspase-Glo 9 reagent was added to each well and gently vortexed for 30 s. After 30 min incubation at room temperature, luminescence was measured against a blank containing all reagents except cells in a microplate reader (Synergy 2, BioTek Instruments, Inc., Winooski, VT, USA). Data are expressed as a percentage of the control cells.

### Apoptosis analysis in the flow cytometer

The number of apoptotic cells was analysed using a cytometric method and double labelling with annexin V/FITC and propidium iodide (PI). This staining allows the classification of cells into viable cells with intact cell membranes (FITC-PI-), early apoptotic cells (FITC + PI-) and late apoptotic/necrotic cells (FITC + PI +).

CHO-K1 cells inoculated into a 6-well plate were treated with 100 μM VOSO_4_ in the presence of 8 mM sodium pyruvate for 24 h. The apoptosis assay was performed using the Annexin V-FITC Apoptosis Assay Kit according to the kit manufacturer’s instructions. Cells were harvested, washed with cold DPBS and centrifuged (200 × g for 10 min). The cell pellet was resuspended in 100 μl ice-cold 1X Binding Buffer and incubated with 5 μl Annexin V-FITC and 5 μl propidium iodide staining solutions for 10 min in the dark. Then, 250 μL of 1X Binding Buffer was added and analysis was performed in a flow cytometer (BD FACSCalibur flow cytometer, 1 Becton Drive Franklin Lakes, NJ, USA). The experiment was performed in two independent replicates.

### Statistical analysis

The data were analysed using the Statistical Package for the Social Sciences (IBM Corp. Version 2020. IBM SPSS Statistics for Windows, version 27.0. Armonk, NY: IBM Corp). The data were checked for outliers using Tukey’s fence. Normal distribution was tested using the Shapiro–Wilk test. One-way ANOVA analysis with Tukey’s post hoc test was performed to assess differences between treatment groups (control, pyruvate, VOSO_4_ and pyruvate + VOSO_4_).

## Results

### Pyruvate reduces the cytotoxicity induced by VOSO_4_in CHO-K1 and NIH/3T3 cell cultures (resazurin-based assay)

**Fig. 1:**
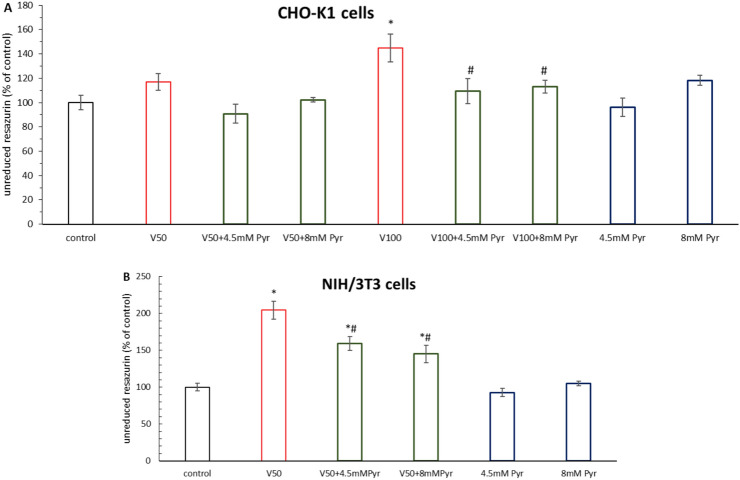
Effects of pyruvate (Pyr) on VOSO_4_-induced cytotoxicity in CHO-K1 (**A**) and NIH/3T3 cells (**B**) measured by resazurin-based assay. The absorbance of the control cells was considered 100%. The bars represent the mean ± SD of two experiments with triplicate determinations of each data point—the larger the bar, the higher the cytotoxicity. * indicates p < 0.001, versus the control group, # indicates p < 0.05 versus the VOSO_4_ -treated group. Data were analysed by one-way ANOVA followed by Tukey’s post hoc test.

The results of the resazurin assay showed that the viability of CHO-K1 cells after 24 h incubation with 4.5 mM and 8 mM pyruvate (Pyr) was comparable to the control (Fig. 1A). Similarly, incubation of cells with 50 μM VOSO_4_ alone or in the 4.5 mM Pyr + 50 μM VOSO_4_ and 8 mM Pyr + 50 μM VOSO_4_ groups did not result in significant changes in cytotoxicity compared to the control. In contrast, incubation with 100 μM VOSO_4_ induced a cytotoxicity of 144.8% compared to the control (p < 0.001). In the 4.5 mM Pyr + 100 μM VOSO_4_ and 8 mM Pyr + 100 μM VOSO_4_ groups, cytotoxicity was reduced to 109.2% (p < 0.01) and 113% (p < 0.05), respectively, which was significant compared to the 100 μM VOSO_4_ group.

The viability of NIH/3T3 cells after 48 h incubation with 4.5 mM and 8 mM pyruvate was comparable to the control (Fig. 1B). Furthermore, 48 h incubation with 50 μM VOSO_4_ induced a cytotoxicity of 204.6% compared to the control (p < 0.001). In contrast, cytotoxicity of 159.3% and 145.2% was observed in cultures concurrently incubated with 50 μM VOSO_4_ + 4.5 mM Pyr and 50 μM VOSO_4_ + 8 mM Pyr, respectively, which was statistically significantly lower than 50 μM VOSO_4_ alone (P < 0.001).

### Phase contrast microscopy

**Fig. 2:**
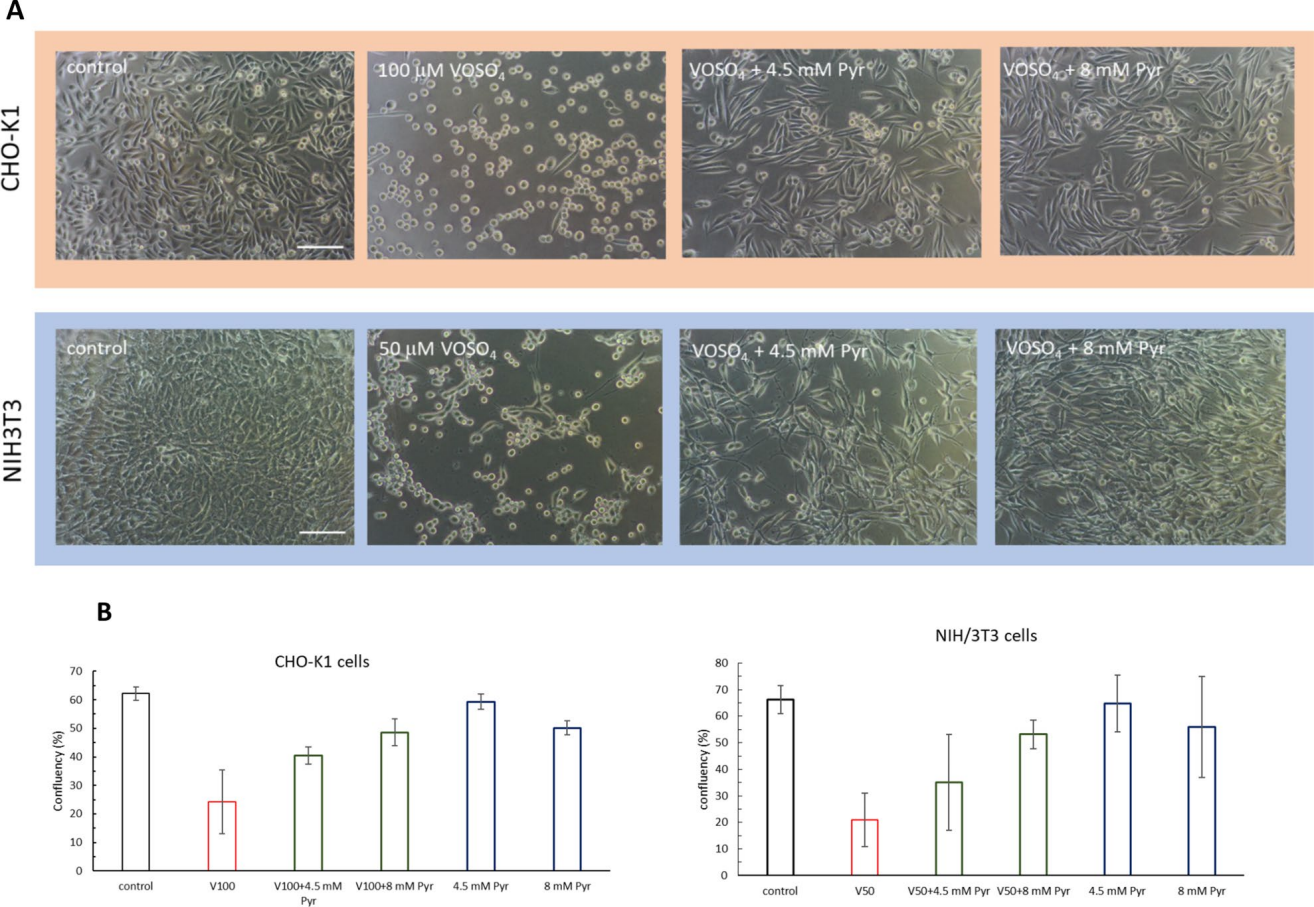
Effects of pyruvate (Pyr) on VOSO_4_-induced cytotoxicity in CHO-K1 and NIH/3T3 cell cultures after 24 h (CHO-K1) or 48 h (3T3) incubation with VOSO_4_ or VOSO4 + Pyr. **A**) Morphology of CHO-K1 and NIH/3T3 cells under phase-contrast microscope, (scale bar = 100 μM); **B**) Percentage of area covered by CHO-K1 and NIH/3T3 cell monolayer calculated using ImageJ.

The protective effect of pyruvate against vanadium cytotoxicity in cell cultures of the CHO-K1 and NIH/3T3 lines was also confirmed by phase-contrast microscopy, and these results were consistent with those of the resazurin assay. Control cells (Fig. 2) and cells incubated with 4.5 mM or 8 mM pyruvate (data not shown) from both cell lines formed a dense monolayer of cells, with the typical morphology for adhered cells. In contrast, in cultures treated with VOSO_4_ alone, the cell density of CHO-K1 and NIH/3T3 cells was significantly reduced compared to the respective control, and most cells had a rounded or spindle shape. For both cell lines, the culture densities of the VOSO_4_ + 4.5 mM Pyr and VOSO_4_ + 8mMPyr groups were significantly higher compared to VOSO_4_ alone. Furthermore, the shape of CHO-K1 and NIH/3T3 cells from the VOSO_4_ + 4.5 mM Pyr and VOSO_4_ + 8mMPyr groups was mostly similar to that of the control cells of each cell line.

### Pyruvate prevented the VOSO_4_-induced decrease in intracellular ATP levels

**Fig. 3:**
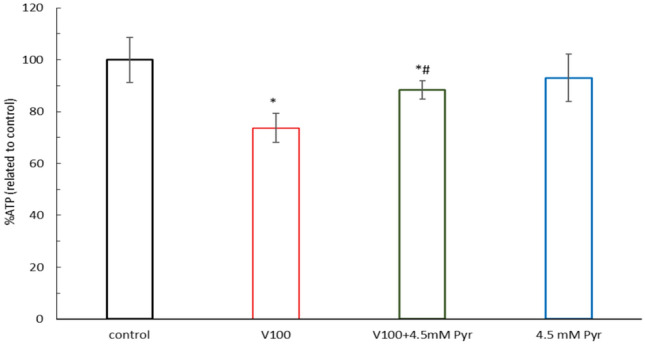
Effects of vanadium (V) and/or pyruvate (Pyr) on ATP level in CHO-K1 cells detected with the luminescent ATP detection method. The CHO-K1 cells were treated with 100 μM VOSO_4_ in the presence or absence of 4.5 mM Pyruvate for 24 h. The luminescence of the control cells was considered 100%. Data represent the mean ± SD of two distinct experiments each performed with sixplicate determinations of each data point, * indicates p < 0.001, versus the control group, # indicates p < 0.05 versus the VOSO_4_ -treated group. Data were analysed by one-way ANOVA followed by Tukey’s post hoc test.

Figure 3 shows the effect of 24 h incubation of CHO-K1 cells with 100 μM VOSO_4_, 4.5 mM Pyr and 100 μM VOSO_4_ + 4.5 mM Pyr on cellular ATP levels. The results showed that ATP levels in the Pyr alone and VOSO_4_ alone groups were 93% (not significant) and 73.7% (p < 0.001), respectively, relative to the control. However, ATP levels in the VOSO_4_ + Pyr group increased significantly compared to VOSO_4_ alone, reaching 88.3%. (P < 0.001).

### Effect of pyruvate and VOSO_4_ on mitochondrial ROS generation

**Fig. 4:**
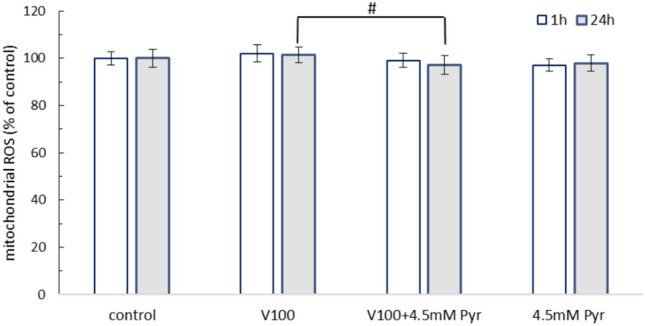
Effects of 100 μM VOSO_4_ and/or 4.5 mM pyruvate (Pyr) on mitochondrial ROS level in CHO-K1 cells. The CHO-K1 cells preloaded with mitochondrial ROS probe (Cayman) were treated with 100 μM VOSO_4_ in the presence or absence of 4.5 mM Pyr for 1 or 24 h. The fluorescence of the control cells was considered 100%. Data represent the mean ± SD of two distinct experiments each performed with sixplicate determinations of each data point. # indicates p < 0.05 versus the VOSO_4_ -treated group. Data were analysed by one-way ANOVA followed by Tukey’s post hoc test.

The effect of 1 h and 24 h incubation of CHO-K1 cells with 100 μM VOSO_4_, 4.5 mM Pyr and 100 μM VOSO_4_ + 4.5 mM Pyr on the level of mitochondrial ROS (Cayman’s reagent) is shown in Fig. 4. In cells exposed to VOSO_4_, VOSO_4_ + Pyr and Pyr alone, ROS production was comparable to the control. However, the level of mitochondrial ROS was lower in the VOSO_4_ + Pyr group compared to VOSO_4_ alone after 1 h (P = 0.084) and especially after 24 h treatment (p = 0.032) with the tested compounds. Results obtained using MitoSOX Red staining showed that the fluorescence was not altered following treatment with 100 µM VOSO₄ or 100 µM VOSO₄ + 4.5 mM Pyr (Supplementary Fig. S1).

### Pyruvate prevented the VOSO_4_-induced decrease in GSH

**Fig. 5:**
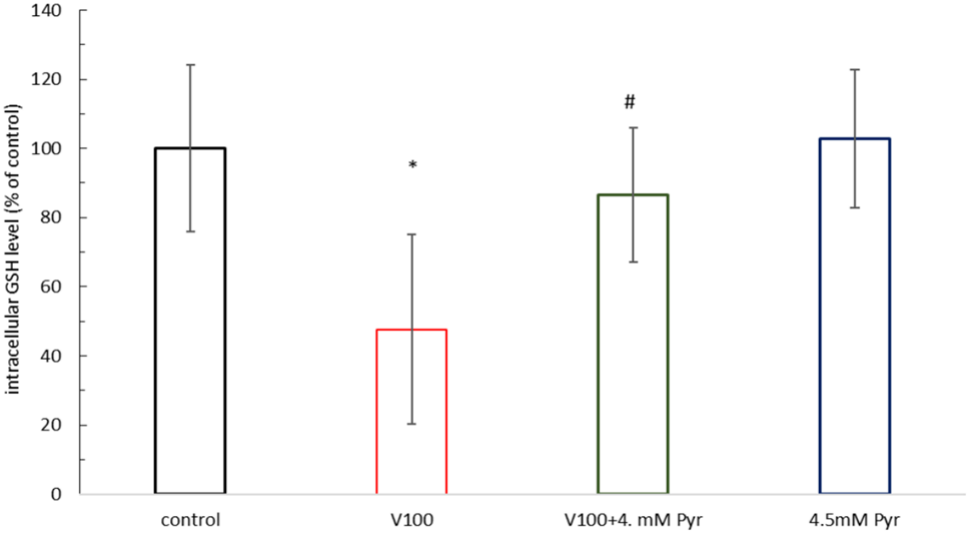
Effects of 100 μM VOSO_4_ and/or 4.5 mM pyruvate (Pyr) on GSH level in CHO-K1 cells. The CHO-K1 cells were treated with 100 μM VOSO_4_ in the presence or absence of 4.5 mM Pyr for 24 h. The GSH level in control cells was taken as 100%. Data represent the mean ± SD of two experiments each carried out in four replicates, * indicates p < 0.001, versus the control group, # indicates p < 0.05 versus the VOSO_4_ -treated group. Data were analysed by one-way ANOVA followed by Tukey’s post hoc test.

The results showed that 24 h exposure of CHO-K1 cells to 100 μM VOSO_4_ caused a significant decrease in intracellular glutathione levels of up to 47% compared to the control (Fig. 5). At the same time, in cells co-exposed to 100 μM VOSO_4_ and 4.5 mM Pyr, glutathione levels were significantly higher than in cells exposed to VOSO_4_ alone, reaching 86.6% compared to control. Treatment of CHO-K1 cells with 4.5 mM Pyr alone did not result in any changes in glutathione levels in the cells tested.

### Effect of pyruvate and VOSO_4_ on caspase-3/7 and caspase-9 activity

**Fig. 6:**
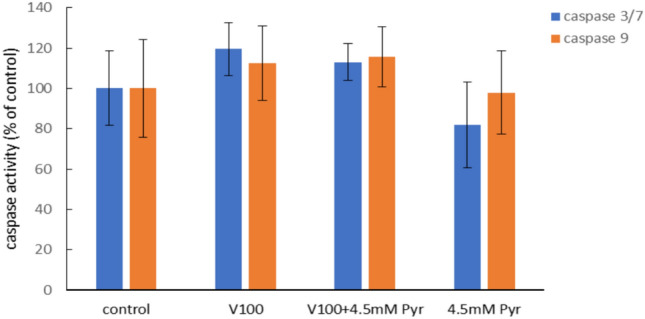
Effects of vanadium (V) and/or pyruvate (Pyr) on caspase 3/7 and caspase 9 activity in CHO-K1 cells detected with the luminescent detection method. The luminescence of the control cells was considered 100%. Data represent the mean ± SD of two experiments each carried out in five replicates.

Incubation of CHO-K1 cells with 100 μM VOSO_4_ resulted in a slight increase in caspase-3/7 and caspase-9 activity of up to 119.5% and 112.3%, respectively compared to control, which however was not statistically significant (Fig. 6). The addition of 4.5 mM Pyr to cells treated with 100 μM VOSO_4_ (VOSO_4_ + Pyr group) had no effect on caspase-3/7 and caspase-9 activity compared to VOSO_4_ alone. Incubation of CHO-K1 cells with 4.5 mM Pyr alone had no effect on caspase-3/7 and −9 activity in the cells tested.

### Effect of pyruvate and VOSO_4_on mode of cell death

**Fig. 7:**
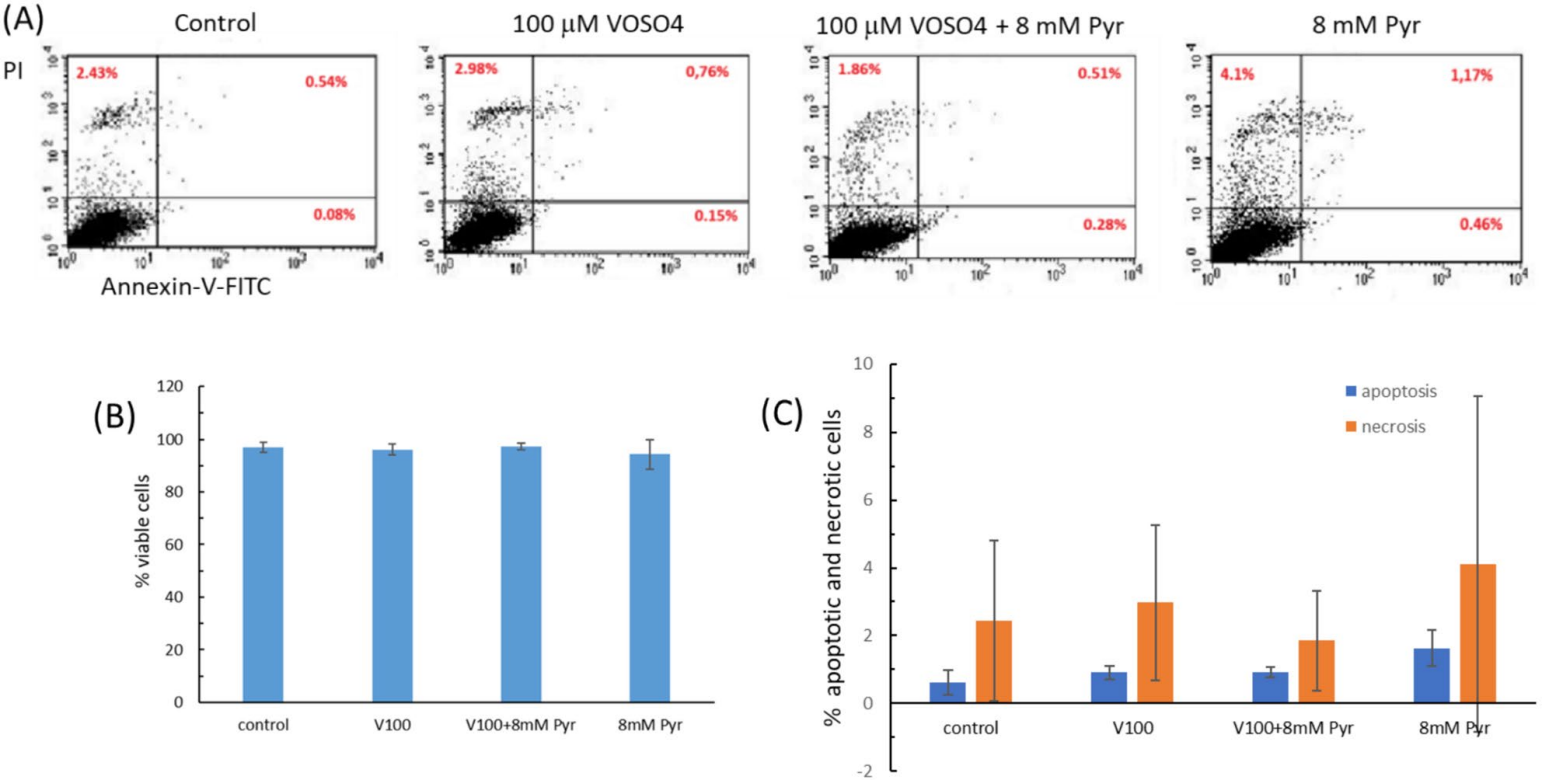
Effects of 100 μM VOSO_4_ and/or 8 mM pyruvate (Pyr) on apoptosis and necrosis induction in CHO-K1 cells. The CHO-K1 cells were treated with 100 μM VOSO_4_ in the presence or absence of 8 mM Pyr for 24 h, double-stained with Annexin-V/FITC and propidium iodide (PI) and analysed by flow cytometer. The total number of cells analyzed for each condition was 10 000. (**A**) The dot plots show one of two independent experiments. (i) Lower left quadrant shows live cells (do not bind annexin and do not stain PI), (ii)Lower right quadrant represents early apoptotic cells (bind annexin only), (iii)Upper right quadrant indicates late apoptotic cells (bind annexin and PI), (iiii) upper left quadrant indicates dead cells (bind PI only). Bar graphs represent the mean ± SD of two independent experiments : (**B**) percentage of living cells, (**C**) apoptotic (early and late) and necrotic cells.

To analyse the effect of VOSO_4_ and pyruvate on the percentage of apoptotic cells, CHO-K1 cells were incubated for 24 h with 100 μM VOSO_4_ without or in the presence of 8 mM Pyr, stained with Annexin V/FITC and propidium iodide and analysed in a flow cytometer (Fig. 7). The results showed that the percentage of CHO-K1 cells in the apoptotic stage was slightly higher in cultures treated with VOSO_4_ alone or VOSO_4_ + Pyr than in the control culture. At the same time, there was no difference in the number of apoptotic cells between cultures exposed to VOSO_4_ alone and VOSO_4_ + Pyr. The number of necrotic cells was slightly higher in cultures with VOSO_4_ alone compared to the control, while cultures incubated with VOSO_4_ + Pyr had a lower percentage of necrotic cells compared to VOSO_4_ alone and the control culture. Interestingly, the analysis showed an increase in the percentage of apoptotic and necrotic cells in the culture treated with 8 mM Pyr alone.

## Discussion

Vanadium is recognised as a toxic metal which, as a component of particle matter (PM2.5), is part of the environmental pollution that is associated with adverse effects on human health^1^. Exogenous pyruvate, on the other hand, is an emerging antioxidant that has been shown in some studies to be effective in ameliorating pathological changes, including those induced by metals, in different cells and tissues^31–33^. However, to date, most of these studies have evaluated the protective effects of ethyl pyruvate, which is not necessarily an effective form of exogenous pyruvate, as ethyl pyruvate cannot be converted to free pyruvate in human plasma^24^. At the same time, there is very little information on the cytoprotective properties of the non-esterified (free) form of pyruvate, i.e. sodium pyruvate, making it difficult to draw definitive conclusions about the cytoprotective properties of exogenous pyruvate. Therefore, in the present study, we investigated the effect of sodium pyruvate at two concentrations of 4.5 and 8 mM on vanadium cytotoxicity using two rodent cell lines, CHO-K1 and NIH/3T3, and then assessed oxidative stress, antioxidant and apoptosis parameters to verify the cytoprotective properties of the free form of pyruvate.

Viability analysis using the resazurin assay confirmed the toxicity of VOSO_4_ at 50 and 100 μM against CHO-K1 and NIH/3T3 cell lines and the protective effect of 4.5 and 8 mM sodium pyruvate against vanadium toxicity (Fig. 1). The cytotoxicity assay results correlated well with microscopic observations, which showed a markedly improved morphology and increased cell confluency of CHO-K1 and NIH/3T3 cells co-administered with pyruvate and VOSO_4_ compared to cells treated with VOSO_4_ alone (Fig. 2). These results were consistent with our previous studies confirming the constancy of cytoprotective properties of sodium pyruvate against vanadium^30^.

Oxidative stress and apoptosis has been implicated in vanadium cytotoxicity in animal cells. Indeed, many previous studies have reported vanadium-dependent induction of cellular and mitochondrial ROS generation, decreased GSH levels, mitochondrial swelling and release of cytochrome c, which initiates the apoptotic cell death pathway^30,34–36^. In addition, vanadium-dependent reductions in cellular ATP levels^37^, of which mitochondria are the main producers in cells (via oxidative phosphorylation)^38^, have been reported by researchers. Our study reported a negative effect of VOSO_4_ on cellular ATP levels compared to the control. The decrease in ATP levels indicates a vanadium-induced dysfunction of ATP synthesis in the cell. Hosseini et al.^35^ observed that the toxic effects of vanadium on protein complexes of the electron transport chain of mitochondria might be responsible for the vanadium-dependent decrease in ATP levels in rat liver cells. In addition, glutathione, the major non-protein thiol in cells, plays an antioxidant role by, among other things, directly reducing ROS or acting as a substrate for antioxidant enzymes such as glutathione peroxidase and glutathione-S- transferase^39^. In addition, reduced glutathione may play a role in the biological detoxification of vanadium, being responsible for the reduction of vanadium(V) to vanadium(IV) and binding reduced vanadium in complexes^5^. In line with the predictions and results of other authors^40,41^, we also observed significantly reduced GSH levels in CHO-K1 cells incubated with vanadium in our study. However, unlike the findings of other researchers^35,42^, vanadium did not affect ROS production in mitochondria in our study. We cannot currently explain this discrepancy, but it may be due to different experimental conditions, including the use of a different research model.

Coincubation of cells with sodium pyruvate partially prevented the vanadium-dependent decrease in intracellular GSH, thereby enhancing cellular antioxidant defences in CHO-K1 cells. Studies by other authors confirm that pyruvate, through direct neutralisation of peroxides, indirectly influenced the maintenance of adequate GSH levels in, among others, astrocytes^43^ and cardiac myocytes^44^. Furthermore, the antioxidant activities of pyruvate are complemented by its role as a metabolic substrate for ATP synthesis in mitochondria^45^. In our study, exogenous pyruvate partially blocked the vanadium-dependent decrease in intracellular ATP levels, thus providing the energy levels necessary to maintain cellular function^46^. The results regarding the effect of vanadium and pyruvate interaction on ATP and GSH were consistent with the resazurin assay and microscopic observation, confirming that ATP and GSH can significantly contribute to the cytoprotective effects of pyruvate.

To assess whether pyruvate reduces vanadium cytotoxicity by inhibiting apoptosis and necrosis, we performed analysis of initiator caspase-9 activity, executioner caspase-3 activity, and quantitative analysis with annexin V-FITC/propidium iodide staining. In our study, apoptosis was not significantly induced by incubation of CHO-K1 cells with VOSO_4_ alone (100 μM) as only a slight, non-significant increase in caspase-3 and caspase-9 activity was observed. In accordance cytometric analysis of the cells using the annexin v-FITC/PI assay showed that vanadium at the concentration tested induced a slight increase in the number of apoptotic and necrotic cells. At the same time, co-incubation of VOSO_4_ with pyruvate did not alter caspase 3 and 9 activity compared to cells incubated with VOSO_4_ alone. Consistent with this finding, the number of apoptotic cells in Annexin V-FITC staining was similar in VOSO_4_ and VOSO_4_ + pyruvate cultures. At the same time, a slight reduction in the number of necrotic cells was observed in cultures treated with VOSO_4_ in the presence of pyruvate. Collectively, exogenous pyruvate appears to inhibit the cytotoxicity of the tested dose of vanadium in the CHO-K1 cells, mainly by the reduction of the necrosis effect.

Sodium pyruvate concentrations showing protective effects in in vitro studies by other authors were mostly in the range of 1–10 mM, which is consistent with the results obtained in our study^19,25,47^. As reported in the in vivo data, the pyruvate concentration in human blood after intravenous administration of sodium pyruvate increased fivefold, i.e. from 0.15 mM to 0.8 mM^48^. Similarly, in goat studies, intravenous administration of sodium pyruvate increased plasma pyruvate from 0.2 mM to 0.5–0.6 mM, while exerting a cardioprotective and anti-inflammatory effect^49^. Previous studies suggest that a plasma pyruvate concentration of at least 1 mM is a concentration that can effectively protect against ROS such as toxic H_2_O_2_ concentrations^24^. As already mentioned, vanadium induces pro-oxidant effects in in vitro cells and animal tissues^50^. Based on the results, the tested sodium pyruvate appears to be a promising cytoprotective antioxidant against vanadium, especially since the safety and some protective aspects of exogenous pyruvate have already been studied in humans^51–53^.

In conclusion, the results suggest that sodium pyruvate has a protective effect against vanadium cytotoxicity in rodent cell line models. The cytoprotective effect of pyruvate has been shown to be mediated by fostering ATP production and antioxidant defences, as well as by inhibiting cellular necrosis. As there is currently no effective antioxidant recommended for the treatment of vanadium poisoning, the exogenous pyruvate tested in the present study, due to its cytoprotective effects, appears to be a promising choice.

## Supplementary Information

Supplementary Information.

## Data Availability

The datasets used and/or analyzed during the current study are available from the corresponding author upon request.

## References

[1] Patel, M. M. et al. Ambient metals, elemental carbon, and wheeze and cough in New York city children through 24 months of age. *Am. J. Respir. Crit. Care Med.* **180**(11), 1107–1113. 10.1164/rccm.200901-0122OC (2009). 19745205 10.1164/rccm.200901-0122OC PMC2784415

[2] Watt, J. A. J. et al. Vanadium: A re-emerging environmental hazard. *Environ. Sci. Technol.* **52**(21), 11973–11974. 10.1021/acs.est.8b05560 (2018). 30358993 10.1021/acs.est.8b05560

[3] Yang, J. et al. Current status and associated human health risk of vanadium in soil in China. *Chemosphere* **171**, 635–643. 10.1016/j.chemosphere.2016.12.058 (2017). 28056450 10.1016/j.chemosphere.2016.12.058

[4] Schlesinger, W. H., Klein, E. M. & Vengosh, A. Global biogeochemical cycle of vanadium. *Proc. Natl. Acad. Sci. U. S. A.* **114**(52), E11092–E11100. 10.1073/pnas.1715500114 (2017). 29229856 10.1073/pnas.1715500114 PMC5748214

[5] Baran, E. J. Vanadium detoxification: Chemical and biochemical aspects. *Chem. Biodivers.* **5**(8), 1475–1484. 10.1002/cbdv.200890136 (2008). 18729109 10.1002/cbdv.200890136

[6] Rehder, D. Vanadium. Its role for humans. *Met. Ions Life Sci.* **13**, 139–169. 10.1007/978-94-007-7500-8_5 (2013). 24470091 10.1007/978-94-007-7500-8_5 PMC7120733

[7] Woodin, M. A. et al. Acute respiratory symptoms in workers exposed to vanadium-rich fuel-oil ash. *Am. J. Ind. Med.* **37**(4), 353–363. 10.1002/(sici)1097-0274(200004)37:4%3c353::aid-ajim5%3e3.0.co;2-l (2000). 10706747 10.1002/(sici)1097-0274(200004)37:4<353::aid-ajim5>3.0.co;2-l

[8] Li, H. et al. Vanadium exposure-induced neurobehavioral alterations among Chinese workers. *Neurotoxicology* **36**, 49–54. 10.1016/j.neuro.2013.02.008 (2013). 23500660 10.1016/j.neuro.2013.02.008 PMC4160152

[9] Prokopciuk, N. et al. The incidence of upper respiratory infections in children is related to the concentration of vanadium in indoor dust aggregates. *Front. Public Health* **12**, 1339755. 10.3389/fpubh.2024.1339755 (2024). 38577275 10.3389/fpubh.2024.1339755 PMC10993999

[10] Hu, J. et al. Effects of trimester-specific exposure to vanadium on ultrasound measures of fetal growth and birth size: A longitudinal prospective prenatal cohort study. *Lancet Planet. Health* **2**(10), e427–e437. 10.1016/S2542-5196(18)30210-9 (2018). 30318100 10.1016/S2542-5196(18)30210-9

[11] Jiang, S. et al. Concentrations of vanadium in urine with hypertension prevalence and blood pressure levels. *Ecotoxicol. Environ. Saf.* **213**, 112028. 10.1016/j.ecoenv.2021.112028 (2021). 33607335 10.1016/j.ecoenv.2021.112028

[12] IARC, International Agency for Research on Cancer. Cobalt in Hard Metals and Cobalt Sulfate, Gallium Arsenide, Indium Phosphide and Vanadium Pentoxide. *Int. Agency Res. Cancer.* **86**, 1 (2006). PMC4781610 16906675

[13] Cuesta, S., Francés, D. & García, G. B. ROS Formation and Antioxidant Status in Brain Areas of Rats Exposed to Sodium Metavanadate. *Neurotoxicol. Teratol.* **33**(2), 297–302. 10.1016/j.ntt.2010.10.010 (2011). 21056100 10.1016/j.ntt.2010.10.010

[14] He, X. et al. Low-Dose vanadium pentoxide perturbed lung metabolism associated with inflammation and fibrosis signaling in male animal and in vitro models. *Am. J. Physiol.-Lung Cell. Mol. Physiol.* **325**(2), L215–L232 (2023). 37310758 10.1152/ajplung.00303.2022 PMC10396228

[15] Tu, W. et al. Vanadium exposure exacerbates allergic airway inflammation and remodeling through triggering reactive oxidative stress. *Front. Immunol.* **13**, 1099509. 10.3389/fimmu.2022.1099509 (2022). 36776398 10.3389/fimmu.2022.1099509 PMC9912158

[16] Zwolak, I. Vanadium carcinogenic, immunotoxic and neurotoxic effects: A review of in vitro studies. *Toxicol. Mech. Methods* **24**(1), 1–12. 10.3109/15376516.2013.843110 (2014). 24147425 10.3109/15376516.2013.843110

[17] Hosseini, M.-J., Seyedrazi, N., Shahraki, J. & Pourahmad, J. Vanadium induces liver toxicity through reductive activation by glutathione and mitochondrial dysfunction. *Adv. Biosci. Biotechnol.* **3**, 1096–1103. 10.4236/abb.2012.38134 (2012).

[18] Zwolak, I. Protective effects of dietary antioxidants against vanadium-induced toxicity: A review. *Oxid. Med. Cell. Longev.* **2020**, 1490316. 10.1155/2020/1490316 (2020). 31998432 10.1155/2020/1490316 PMC6973198

[19] Poteet, E. et al. In vitro protection by pyruvate against cadmium-induced cytotoxicity in hippocampal HT-22 cells. *J. Appl. Toxicol. JAT* **34**(8), 903–913. 10.1002/jat.2913 (2014). 24037965 10.1002/jat.2913

[20] Zhao, Z. et al. Sodium pyruvate exerts protective effects against cigarette smoke extract-induced ferroptosis in alveolar and bronchial epithelial cells through the GPX4/Nrf2 Axis. *J. Inflamm. Lond. Engl.* **20**(1), 28. 10.1186/s12950-023-00347-w (2023). 10.1186/s12950-023-00347-w PMC10441695 37605161

[21] Kim, Y.-M., Choi, S. Y., Hwang, O. & Lee, J.-Y. Pyruvate prevents dopaminergic neurodegeneration and motor deficits in the 1-Methyl-4-Phenyl-1,2,3,6-Tetrahydropyridine model of Parkinson’s disease. *Mol. Neurobiol.* **59**(11), 6956–6970. 10.1007/s12035-022-03017-9 (2022). 36057709 10.1007/s12035-022-03017-9

[22] Varma, S. D. & Chandrasekaran, K. High sugar-induced repression of antioxidant and anti-apoptotic genes in lens: Reversal by pyruvate. *Mol. Cell. Biochem.* **403**(1–2), 149–158. 10.1007/s11010-015-2345-y (2015). 25711401 10.1007/s11010-015-2345-y

[23] Giandomenico, A. R., Cerniglia, G. E., Biaglow, J. E., Stevens, C. W. & Koch, C. J. The importance of sodium pyruvate in assessing damage produced by hydrogen peroxide. *Free Radic. Biol. Med.* **23**(3), 426–434. 10.1016/s0891-5849(97)00113-5 (1997). 9214579 10.1016/s0891-5849(97)00113-5

[24] Guarino, V. A., Oldham, W. M., Loscalzo, J. & Zhang, Y.-Y. Reaction rate of pyruvate and hydrogen peroxide: Assessing antioxidant capacity of pyruvate under biological conditions. *Sci. Rep.* **9**(1), 19568. 10.1038/s41598-019-55951-9 (2019). 31862934 10.1038/s41598-019-55951-9 PMC6925109

[25] Ramos-Ibeas, P., Barandalla, M., Colleoni, S. & Lazzari, G. Pyruvate antioxidant roles in human fibroblasts and embryonic stem cells. *Mol. Cell. Biochem.* **429**(1–2), 137–150. 10.1007/s11010-017-2942-z (2017). 28247212 10.1007/s11010-017-2942-z

[26] Battaglia, S. et al. Uridine and pyruvate protect T cells’ proliferative capacity from mitochondrial toxic antibiotics: A clinical pilot study. *Sci. Rep.* **11**(1), 12841. 10.1038/s41598-021-91559-8 (2021). 34145306 10.1038/s41598-021-91559-8 PMC8213784

[27] Kang, Y. H., Chung, S. J., Kang, I. J., Park, J. H. & Bünger, R. Intramitochondrial pyruvate attenuates hydrogen peroxide-induced apoptosis in bovine pulmonary artery endothelium. *Mol. Cell. Biochem.* **216**(1–2), 37–46. 10.1023/a:1011040026620 (2001). 11216862 10.1023/a:1011040026620

[28] Wang, X. et al. Pyruvate protects mitochondria from oxidative stress in human neuroblastoma SK-N-SH cells. *Brain Res.* **1132**(1), 1–9. 10.1016/j.brainres.2006.11.032 (2007). 17174285 10.1016/j.brainres.2006.11.032 PMC1853247

[29] Zwolak, I. & Gołębiowska, D. Protective activity of pyruvate against vanadium-dependent cytotoxicity in chinese hamster ovary (CHO-K1) cells. *Toxicol. Ind. Health* **34**(5), 283–292. 10.1177/0748233718754979 (2018). 29529943 10.1177/0748233718754979

[30] Zwolak, I. & Wnuk, E. Effects of sodium pyruvate on vanadyl sulphate-induced reactive species generation and mitochondrial destabilisation in CHO-K1 cells. *Antioxid. Basel Switz.* **11**(5), 909. 10.3390/antiox11050909 (2022). 10.3390/antiox11050909 PMC9137755 35624773

[31] Bhat, S. M., Massey, N., Karriker, L. A., Singh, B. & Charavaryamath, C. Ethyl pyruvate reduces organic dust-induced airway inflammation by targeting HMGB1-RAGE signaling. *Respir. Res.* **20**(1), 27. 10.1186/s12931-019-0992-3 (2019). 30728013 10.1186/s12931-019-0992-3 PMC6364446

[32] Chavali, V. D., Agarwal, M., Vyas, V. K. & Saxena, B. Neuroprotective effects of ethyl pyruvate against aluminum chloride-induced alzheimer’s disease in rats via inhibiting Toll-Like receptor 4. *J. Mol. Neurosci. MN* **70**(6), 836–850. 10.1007/s12031-020-01489-9 (2020). 32030557 10.1007/s12031-020-01489-9

[33] Yang, R., Zhu, S. & Tonnessen, T. I. Ethyl pyruvate is a novel anti-inflammatory agent to treat multiple inflammatory organ injuries. *J. Inflamm. Lond. Engl.* **13**, 37. 10.1186/s12950-016-0144-1 (2016). 10.1186/s12950-016-0144-1 PMC5135784 27980458

[34] Aureliano, M., De Sousa-Coelho, A. L., Dolan, C. C., Roess, D. A. & Crans, D. C. Biological consequences of vanadium effects on formation of reactive oxygen species and lipid peroxidation. *Int. J. Mol. Sci.* **24**(6), 5382. 10.3390/ijms24065382 (2023). 36982458 10.3390/ijms24065382 PMC10049017

[35] Hosseini, M.-J., Shaki, F., Ghazi-Khansari, M. & Pourahmad, J. Toxicity of vanadium on isolated rat liver mitochondria: A new mechanistic approach. *Met. Integr. Biometal Sci.* **5**(2), 152–166. 10.1039/c2mt20198d (2013). 10.1039/c2mt20198d 23306434

[36] Hosseini, M.-J. et al. Protective effects of sesamum indicum extract against oxidative stress induced by vanadium on isolated rat hepatocytes. *Environ. Toxicol.* **31**(8), 979–985. 10.1002/tox.22107 (2016). 25727928 10.1002/tox.22107

[37] Li, J.-B. et al. Effects of VO2 nanoparticles on human liver HepG2 cells: Cytotoxicity, genotoxicity, and glucose and lipid metabolism disorders. *NanoImpact* **24**, 100351. 10.1016/j.impact.2021.100351 (2021). 35559810 10.1016/j.impact.2021.100351

[38] Chen, M.-M. et al. Mitochondrial function and reactive oxygen/nitrogen species in skeletal muscle. *Front. Cell Dev. Biol.* **10**, 826981. 10.3389/fcell.2022.826981 (2022). 35265618 10.3389/fcell.2022.826981 PMC8898899

[39] Gaucher, C. et al. Glutathione: Antioxidant properties dedicated to nanotechnologies. *Antioxid. Basel Switz.* **7**(5), 62. 10.3390/antiox7050062 (2018). 10.3390/antiox7050062 PMC5981248 29702624

[40] Folarin, O. R., Adaramoye, O. A., Akanni, O. O. & Olopade, J. O. Changes in the brain antioxidant profile after chronic vanadium administration in mice. *Metab. Brain Dis.* **33**(2), 377–385. 10.1007/s11011-017-0070-9 (2018). 28744799 10.1007/s11011-017-0070-9

[41] Mukhtiar, M. et al. Evaluation of the interaction of vanadium with glutathione in human blood components. *Pak. J. Pharm. Sci.* **25**(3), 549–553 (2012). 22713940

[42] He, X. et al. Vanadium pentoxide induced oxidative stress and cellular senescence in human lung fibroblasts. *Redox Biol.* **55**, 102409. 10.1016/j.redox.2022.102409 (2022). 35870339 10.1016/j.redox.2022.102409 PMC9307685

[43] Miao, Y. et al. Protection by pyruvate against glutamate neurotoxicity is mediated by astrocytes through a glutathione-dependent mechanism. *Mol. Biol. Rep.* **38**(5), 3235–3242. 10.1007/s11033-010-9998-0 (2011). 20182801 10.1007/s11033-010-9998-0

[44] Mallet, R. T. & Sun, J. Antioxidant properties of myocardial fuels. *Mol. Cell. Biochem.* **253**(1–2), 103–111. 10.1023/a:1026009519783 (2003). 14619960 10.1023/a:1026009519783

[45] Crestanello, J. A., Kamelgard, J. & Whitman, G. J. The cumulative nature of pyruvate’s dual mechanism for myocardial protection. *J. Surg. Res.* **59**(1), 198–204. 10.1006/jsre.1995.1154 (1995). 7630128 10.1006/jsre.1995.1154

[46] Ryou, M.-G. et al. Pyruvate minimizes rtPA Toxicity from in vitro oxygen-glucose deprivation and reoxygenation. *Brain Res.* **1530**, 66–75. 10.1016/j.brainres.2013.07.029 (2013). 23891792 10.1016/j.brainres.2013.07.029 PMC4007160

[47] Natoli, R. et al. The role of pyruvate in protecting 661W Photoreceptor-Like cells against light-induced cell death. *Curr. Eye Res.* **41**(11), 1473–1481. 10.3109/02713683.2016.1139725 (2016). 27217092 10.3109/02713683.2016.1139725

[48] Constantin-Teodosiu, D., Simpson, E. J. & Greenhaff, P. L. The importance of pyruvate availability to PDC activation and anaplerosis in human skeletal muscle. *Am. J. Physiol.* **276**(3), E472-478. 10.1152/ajpendo.1999.276.3.E472 (1999). 10070012 10.1152/ajpendo.1999.276.3.E472

[49] Flaherty, D. C. et al. Pyruvate-Fortified fluid resuscitation improves hemodynamic stability while suppressing systemic inflammation and myocardial oxidative stress after hemorrhagic shock. *Mil. Med.* **175**(3), 166–172. 10.7205/milmed-d-09-00161 (2010). 20358705 10.7205/milmed-d-09-00161

[50] Wilk, A., Szypulska-Koziarska, D. & Wiszniewska, B. The toxicity of vanadium on gastrointestinal, urinary and reproductive system, and its influence on fertility and fetuses malformations. *Postepy. Hig. Med. Doswiadczalnej. Online* **71**, 850–859. 10.5604/01.3001.0010.4783 (2017). 10.5604/01.3001.0010.4783 29039350

[51] Fujii, T. et al. Efficacy of pyruvate therapy in patients with mitochondrial disease: A semi-quantitative clinical evaluation study. *Mol. Genet. Metab.* **112**(2), 133–138. 10.1016/j.ymgme.2014.04.008 (2014). 24830361 10.1016/j.ymgme.2014.04.008

[52] Koga, Y., Povalko, N., Inoue, E., Nashiki, K. & Tanaka, M. Biomarkers and clinical rating scales for sodium pyruvate therapy in patients with mitochondrial disease. *Mitochondrion* **48**, 11–15. 10.1016/j.mito.2019.02.001 (2019). 30738201 10.1016/j.mito.2019.02.001

[53] Martin, A., Lupfer, C. & Amen, R. Sodium pyruvate nasal spray reduces the severity of nasal inflammation and congestion in patients with allergic rhinitis. *J. Aerosol Med. Pulm. Drug Deliv.* **35**(6), 291–295. 10.1089/jamp.2022.0025 (2022). 35960504 10.1089/jamp.2022.0025 PMC9807276

