# Supplementary material for: The role of sodium pyruvate in mitigating the cytotoxic effects of vanadium on CHO-K1 cells
Source: Sci Rep. 2025 Jul 5;15:24006. doi: 10.1038/s41598-025-09606-7 (PMC12227759; doi:10.1038/s41598-025-09606-7)
Supplement: Supplementary file 1 — Supplementary Information. [file 41598_2025_9606_MOESM1_ESM.docx]

**The role of sodium pyruvate in mitigating the cytotoxic effects of vanadium on CHO-K1 cells**

**Iwona Zwolak^a*^, Ewa Wnuk^a^, Elżbieta Kochanowicz^b^**

^a^Department of Biomedicine and Environmental Research, Institute of Biological Sciences, Faculty of Medicine, The John Paul II Catholic University of Lublin, Konstantynów Ave. 1J, 20-708 Lublin, Poland

^b^Department of Molecular Biology, Institute of Biological Sciences, Faculty of Medicine, The John Paul II Catholic University of Lublin, Konstantynów Ave. 1J, 20-708 Lublin, Poland ^*^[iwona.zwolak@kul.pl](mailto:iwona.zwolak@kul.pl)


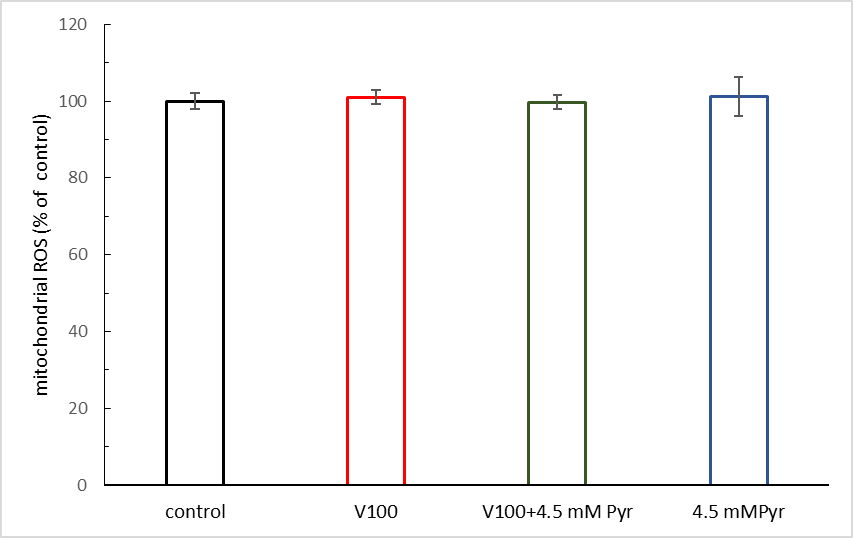


**Supplementary Figure S1**. Effects of 100 μM VOSO_4_ and/or 4.5 mM Pyr following 1 h co-incubation on mitochondrial ROS level in CHO-K1 cells measured using MitoSox Red as a fluorescence indicator (excitation at 360/40 and emission at 590/35 nm). The fluorescence of the control cells was considered 100%. Data represent the mean ± SD of two distinct experiments each performed with sixplicate determinations of each data point.
